# Tumor biology, clinicopathological characteristics and prognosis of screen detected T1 invasive non-palpable breast cancer in asymptomatic Chinese women (2001–2014)

**DOI:** 10.18632/oncotarget.15431

**Published:** 2017-02-17

**Authors:** Bo Pan, Ru Yao, Yi-Dong Zhou, Qing-Li Zhu, Jie Shi, Qian-Qian Xu, Chang-Jun Wang, Shan-Shan You, Feng Mao, Yan Lin, Song-Jie Shen, Zhi-Yong Liang, Yu-Xin Jiang, Qiang Sun

**Affiliations:** ^1^ Department of Breast Surgery, Peking Union Medical College Hospital, Chinese Academy of Medical Sciences and Peking Union Medical College, Beijing, 100730, P. R. China; ^2^ Department of Ultrasound, Peking Union Medical College Hospital, Chinese Academy of Medical Sciences and Peking Union Medical College, Beijing, 100730, P. R. China; ^3^ Department of Pathology, Peking Union Medical College Hospital, Chinese Academy of Medical Sciences and Peking Union Medical College, Beijing, 100730, P. R. China

**Keywords:** non-palpable breast cancer, screen-detected breast cancer, T1, lymph node metastasis, prognosis

## Abstract

**Background:**

Mammography screening usually detects low-risk breast cancer in the western world. However, little is known about the ultrasound and mammography screen-detected T1 invasive non-palpable breast cancer (NPBC) in asymptomatic Chinese women.

**Results:**

With the increase of tumor size (T1a, b, c), lymph node positivity (8.7%, 18.3%, 26.0%, *p* = 0.018), pN (*p* = 0.028) and TNM stage (*p* = 0.035) increased accordingly. Tumor size (T1a, b, c) was correlated with high Ki-67 index (defined as ≥ 14%, 37.9%, 45.8%, 56.2%, *p* = 0.017), chemotherapy (20.4%, 35.2%, 57.3%, *p* < 0.001) and targeted therapy (2.9%, 9.9%, 15.1%, *p* = 0.008). T1a disease had higher chance of being luminal A and accompanied with ductal carcinoma *in situ* (DCIS), while T1c tumor being triple-negative and without DCIS. The 5-year disease free survival (DFS) of T1a, b, c NPBC were 99.0%, 96.9% and 92.9%, whereas the 5-year overall survival (OS) were 100.0%, 100.0% and 97.9% respectively. There was no significant difference in 5-year DFS or OS among the T1 NPBC subgroups or subtypes/immunophenotypes.

**Patients and methods:**

From 2001 to 2014, 4,574 screening positive women received biopsies in Peking Union Medical College (PUMC) Hospital, and 729 NPBC including 437 T1 unilateral invasive NPBC were diagnosed. With a median follow-up time of 32 months (6–163 months), the clinicopathological characteristics, treatment choice, 5-year DFS and OS were compared between T1a, T1b and T1c NPBC. The DFS and OS prognostic factors were identified.

**Conclusion:**

Screen-detected T1 invasive NPBC could be regarded as low-risk cancer in Chinese women. TNM stage and LN metastasis instead of molecular subtype was identified as the DFS prognostic factors while radiotherapy as the OS predictor.

## INTRODUCTION

With the rapid increase of breast cancer incidence in China, little is known yet about the biological behavior, clincopathological characteristics and prognostic factors of screen-detected non-palpable breast cancer (NPBC) in asymptomatic Chinese women. Given the huge population and the diversified modalities of breast cancer screening, the mainstay screening method in China is the hospital-based intentional screening among self-referred asymptomatic women [1–4]. Studies have shown that mammography (MG) usually detects low-risk cancer with concerns of over-diagnosis, and that the tumor stage, molecular subtype and detection mode could help to define low risk patients within the screen detected breast cancer [5–9]. It remains unclear whether this is true for a different ethnicity with distinct cancer epidemiology and complex screening background in China.

In our previous work, we showed with a multi-center randomized controlled trial that ultrasound (US) could detect breast cancer with improved sensitivity and accuracy in high risk Chinese women [10]. Then we demonstrated within a retrospective cohort on a hospital screening basis that US and MG detected NPBC had similar long-term survival [3]. Thus US would not delay the early detection of NPBC compared to MG, and it would be justified to combine US-detected and MG-detected NPBC together for tumor biology analysis. Considering the majority of screen-detected NPBC was T1 tumors, we would like to address the following questions in this study: 1) How is the lymph node (LN) metastasis potential and the subtype/immunophenotype distribution of the T1 invasive NPBC? 2) For these small invasive cancers, is it the TNM stage representing the early detection level that influences the prognosis, or the subtype/immunophenotype revealing the tumor biological nature that does? How do these two key factors play the roles? 3) Is screen-detected T1 invasive NPBC low risk cancer? How to differentiate the individualized risk? With answers to these questions, the understanding of the biological behavior, clinicopathological features and survival of screen-detected breast cancer in Chinese women would be largely improved.

## RESULTS

### Descriptive information of the study cohort

**Figure 1:**
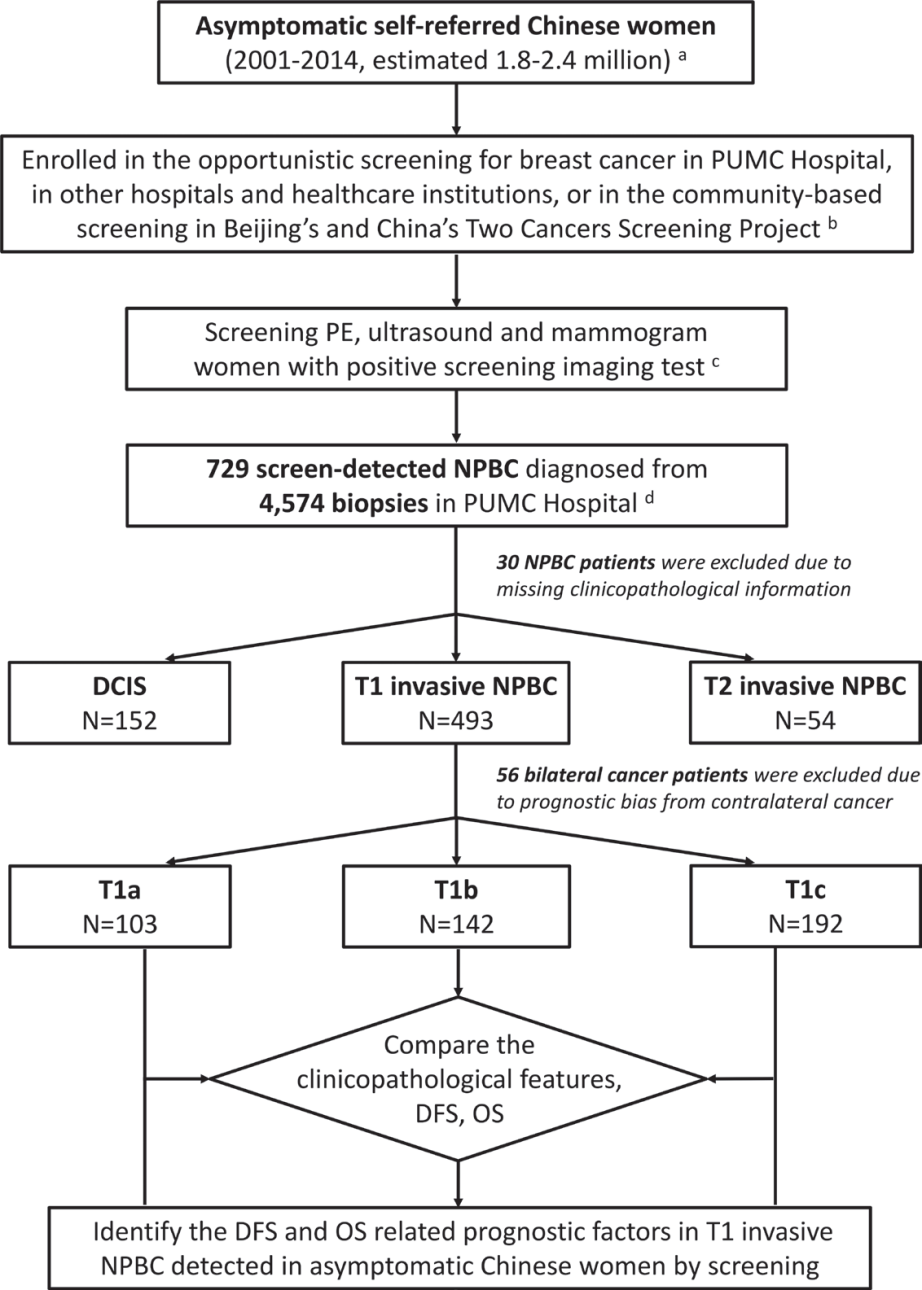
Diagram of the research design The non-palpable breast cancer (NPBC) including T1 invasive NPBC was diagnosed from women with positive screening imaging test detected by both opportunistic screening and community screening with ultrasound and mammography among asymptomatic Chinese women. The clinico-pathological characteristics, disease free survival (DFS) and overall survival (OS) were compared between T1a, T1b, and T1c unilateral invasive NPBC. DFS and OS related prognostic factors of T1 invasive NPBC were identified. **^a^** The total 1.8–2.4 million asymptomatic women participated in the hospital-based screening was estimated with the 729 screen-detected NPBC and the overall incidence of 30–40/ten thousand in China. ^b^ The Beijing's Two Cancers Screening Project had screened breast cancer with physical examination (PE) and ultrasound in a combination of community-based and hospital-based manner. ^c^ Positive imaging tests of US and MG was defined as BI-RADS 4 and 5, whereas negative imaging study was defined as BI-RADS 1, 2 and 3. ^d^ Part of the women with positive screening imaging test (4,574 women) were transferred and treated in PUMC Hospital.

437 T1 unilateral invasive NPBC was included in this study as described in Patients and Methods (Figure 1), comprising 62.5% of the 699 screen-detected NPBC and 5.0% of contemporary 8,821 breast cancer treated in PUMC Hospital. 429 patients (98.2%) were treated during the recent ten years (2005–2014) while 361 patients (82.6%) were treated during the recent five years (2010–2014). 288 patients (65.9%) were pre-menopausal and 149 (34.1%) post-menopausal. With a median follow-up time of 32 months (6–163 months, mean 40 months), 9 patients developed recurrence or metastasis, including 5 local recurrence and 4 distant metastasis. Three patients passed away and one of them was due to breast cancer related death.

### Comparison of clinicopathological characteristics between T1a, T1b and T1c invasive NPBC

In the comparison between T1a, T1b and T1c NPBC, there was no significant difference in age, multi-focal disease, lymphovascular invasion (LVI), ER, PR, Her2, p53, radiotherapy and endocrine therapy (Supplementary Table 1). Mammography (MG) could detect higher percentage of T1a NPBC than of T1b and T1c disease (25.2% vs 7.3–9.5%, *p* < 0.001), because these T1a NPBC was usually the micro-invasive or focal invasive cancer accompanied with DCIS (71.8% vs 8.9–14.1%, *p* < 0.001). With the increase of tumor size (T1a, b, c), lymph node positivity (8.7%, 18.3%, 26.0%, *p* = 0.018), pN (*p* = 0.028) and TNM stage (*p* = 0.035) increased accordingly. Tumor size (T1a, b, c) was also correlated with high Ki-67 index (defined as ≥ 14%, 37.9%, 45.8%, 56.2%, *p* = 0.017) and chances of receiving chemotherapy (20.4%, 35.2%, 57.3%, *p* < 0.001) and targeted therapy. (2.9%, 9.9%, 15.1%, *p* = 0.008). As for the subtype/immunophenotype, the chance of developing luminal A cancer was much lower for T1c compared to T1a, b disease (32.8% vs 40.7–43.7%, *p* = 0.021) whereas chance of developing triple-negative breast cancer (TNBC) much higher (13.0% vs 7.0–7.8%, *p* = 0.039). There was no significant difference among T1a, b, c in percentage of luminal B and Her2 subtype. The breast conserving rate was higher for T1b NPBC compared with T1a or T1c diseases (29.6% vs 13.6 and 19.8%, *p* = 0.008) (Supplementary Table 1).

### Survival outcomes and prognostic factors of T1 invasive NPBC

**Figure 2:**
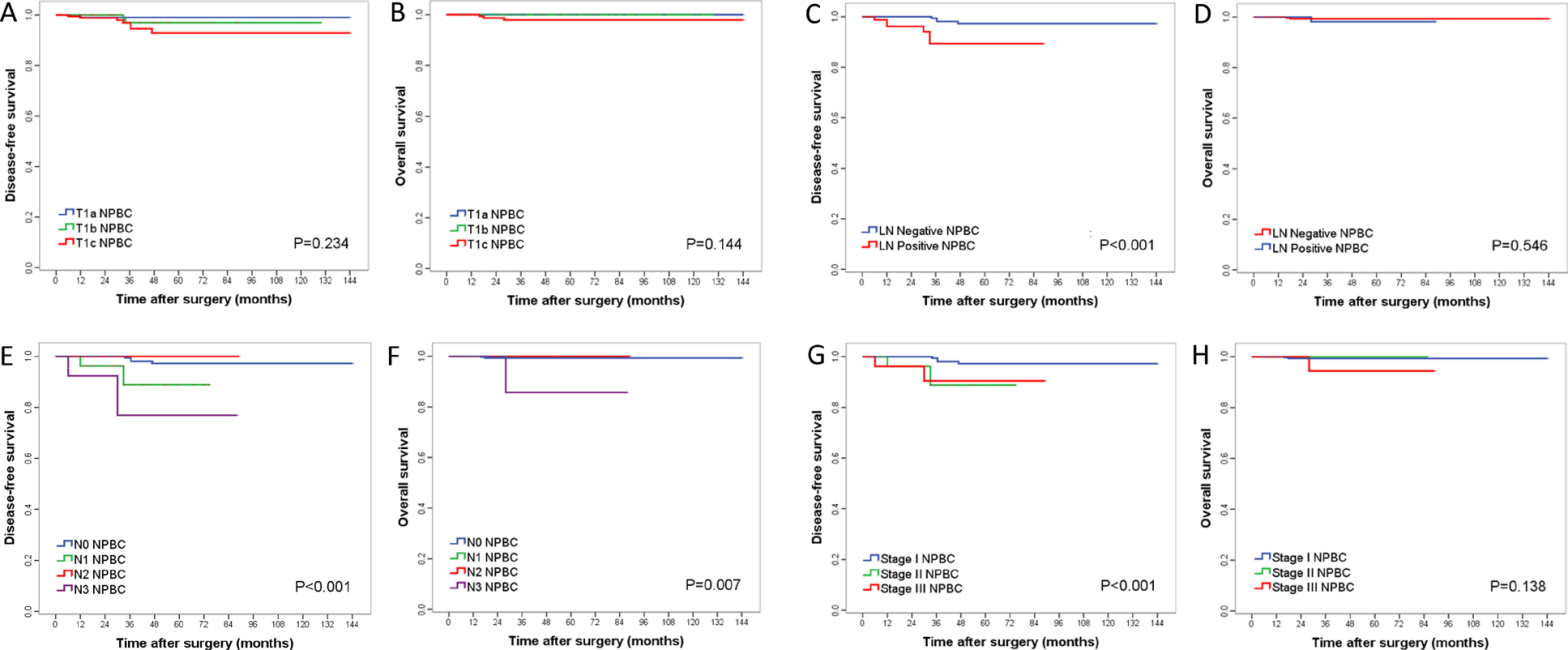
Kaplan–Meier curves for disease-free survival (DFS) and overall survival (OS) among different TNM subgroups of T1 invasive NPBC patients A/B for comparison of T1a, b, c stage invasive NPBC; C/D for LN positive and negative of T1 invasive NPBC; E/F for pN stage of T1 invasive NPBC; G/H for TNM stage of T1 invasive NPBC. *P*-values were calculated using log-rank test.

**Figure 3:**
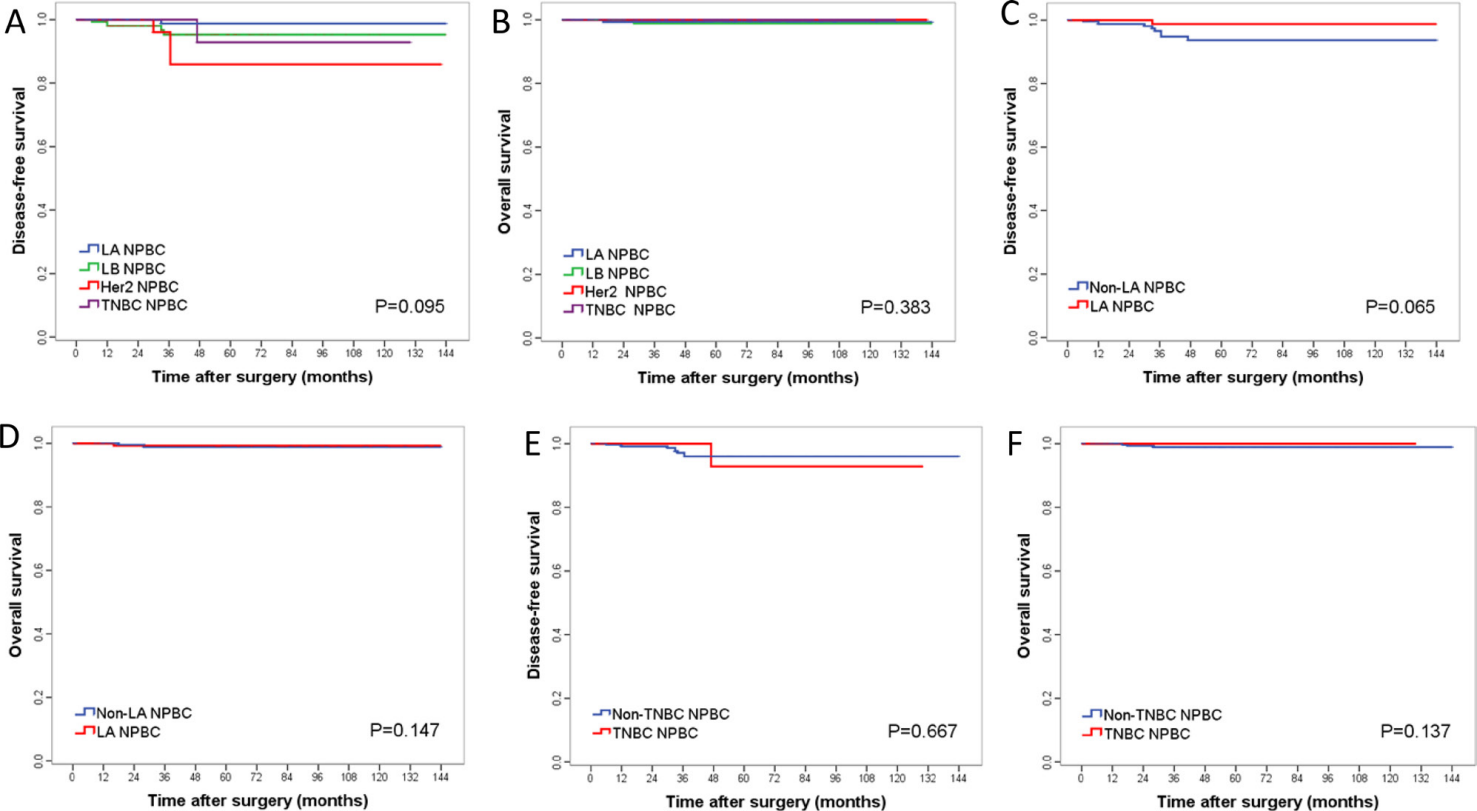
Kaplan–Meier curves for DFS and OS among different subtype/immunophenotype of T1 invasive NPBC patients A/B for all subtypes/immunophenotypes of T1 invasive NPBC; C/D for Luminal A T1 invasive NPBC; E/F for triple-negative T1 invasive NPBC. *P*-values were calculated using log-rank test.

The 5-year Kaplan–Meier estimated disease free survival (DFS) of T1a, T1b and T1c NPBC patients were 99.0%, 96.9% and 92.9%, whereas the 5-year overall survival (OS) were 100.0%, 100.0% and 97.9% respectively. There was no significant difference in 5-year DFS (*p* = 0.234) or OS (*p* = 0.144) between the T1 subgroups of NPBC (Figure 2, Table 1). Nor was there significant difference in DFS (*p* = 0.095) or OS (*p* = 0.383) among subtypes/immunophenotypes, between luminal A vs non-luminal A NPBC, or between TNBC vs non-TNBC (Figure 3, Table 1). There was significant difference in DFS among different LN status (*p* < 0.001), pN stage (*p* < 0.001) and pTNM stage (*p* = 0.002) as well as in OS among different pN stage (*p* = 0.007) (Figure 3, Table 1). For NPBC with high Ki-67 index (≥ 14%), there was no significant difference in DFS (*p* = 0.250) or OS (*p* = 0.403) among pT1a, b, c subgroups (data not shown).

**Table 1:** Comparison of the Kaplan–Meier estimated 5-year DFS and OS (%) among different pT, lymph node status, pN, pTNM stage and molecular subtype in T1 invasive NPBC

TNM (No.)	Subgroup (No.)	5-year DFS (%)	*P* value	5-year OS (%)	*P* value
**pT stage (437)**	T1a (103)	99.0	0.234	100.0	0.144
	T1b (142)	96.9		100.0	
	T1c (192)	92.9		97.9	
**LN status (437)**	LN negative (352)	97.3	**< 0.001**	99.3	0.546
	LN positive (85)	89.4		98.1	
**pN stage (437)**	N0 (352)	97.3	**< 0.001**	99.3	**0.007**
	N1 (58)	88.8		100.0	
	N2 (13)	100.0		100.0	
	N3 (14)	76.9		85.7	
**pTNM stage (437)**	I (353)	97.3	**0.002**	99.3	0.138
	II (57)	88.8		100.0	
	III (27)	90.5		94.4	
**Molecular Subtypes (437)**	LA (167)	98.8	0.095	99.3	0.383
	LB (165)	95.2		98.9	
	Her2 (34)	85.9		100.0	
	TNBC (43)	92.9		100.0	
	Unknown (28)	100.0*		95.2	

Abbreviations: NPBC, non-palpable breast cancer; LN, lymph node; LA, luminal A; LB, luminal B; TNBC, triple-negative breast cancer; DFS, disease free survival; OS, overall survival.

*Among these 28 NPBC of unknown molecular subtype, there were two patients passed away due to other reasons unrelated to breast cancer. So the DFS was actually breast-cancer specific survival.

DFS prognostic factor for T1 NPBC included lymph node status (*p* = 0.043), pN (*p* = 0.000) and TNM stage (*p* = 0.001) by both univariate and multivariate Cox analysis (Table 2). LVI, Ki-67 expression, chemotherapy and targeted therapy was identified as potential DFS factors only by univariate analysis. However, these factors were not significant by multivariate Cox analysis. Conversely, PR status and surgery was identified as potential DFS factors only by multivariate Cox analysis but not univariate analysis (Table 2). Radiotherapy was identified as the OS related predictor by both univariate and multivariate Cox analysis (*p* = 0.045) (Table 3), whereas pN stage, Ki-67 expression as potential OS factors by only univariate analysis. None of the subtype/immunophenotype related factor was identified as T1 invasive NPBC survival factors. The screening method, age distribution, histological grade, pT, ER, Her2, p53, subtype/immunophenotype, endocrine therapy were neither DFS factors nor OS predictors (Tables 2, 3).

**Table 2:** Univariate and multivariate Cox analysis of DFS prognostic factors of screen-detected T1 invasive NPBC patients

Variables	Univariate^a^	Multivariate^b^	
	*P*^c^	HR (95% CI)	*P*^c^
**Age at diagnosis**	0.240	0.833 (0.305, 2.274)	0.722
**Screening method**	0.358	1.821 (0.089, 37.135)	0.697
**Accompanied with DCIS**	0.747	1.13 (0.116, 3.721)	0.909
**Histological grade**	0.412	0.788 (0.173, 3.584)	0.758
**pT**	0.234	9.989 (0.000, 19330375.35)	0.755
**Lymph node status**	**0.000**	**25.1709 (1.108, 571.734)**	**0.043**
**pN**	**0.000**	**3.163 (1.716, 5.832)**	**0.000**
**TNM stage^d^**	**0.000**	**0.000 (0.000, 0.022)**	**0.001**
**Focality**	0.485	0.650 (0.056, 7.566)	0.731
**LVI**	**0.013**	7.023 (0.659, 74.825)	0.106
**ER status**	0.443	1.055 (0.000, 3863.340)	0.990
**PR status**	0.145	**0.204 (0.054, 0.766)**	0.018
**Hormone receptor status**	0.301	0.885 (0.000, 2.698E + 046)	0.998
**HER2 status**	0.126	1.078 (0.040, 28.931)	0.964
**Ki-67 expression**	**0.022**	1.260 (0.036, 44.366)	0.899
**p53**	0.242	0.861 (0.140, 5.310)	0.872
**Immunophenotype**	0.095	1.057 (0.016, 70.346)	0.979
**Luminal A**	0.065	4.978 (0.000, 9.184E + 14)	0.924
**Luminal B**	0.462	0.300 (0.000, 4.635E + 13)	0.942
**HER2**	0.073	8.085 (0.000, 7.317E + 25)	0.943
**TNBC**	0.667	0.688 (0.001, 364.586)	0.907
**Surgery**	0.117	**0.164 (0.036, 0.742)**	0.019
**Chemotherapy**	**0.008**	1.097 (0.081, 14.891)	0.944
**Radiotherapy**	0.271	0.496 (0.019, 12.705)	0.672
**Anti-Her2 targeted therapy**	**0.019**	1.124 (0.040, 31.578)	0.945
**Endocrine therapy**	0.304	1.051 (0.000, 2.107E + 046)	0.999

Abbreviations: NPBC, non-palpable breast cancer; US, ultrasound; MG, mammography; SD, standard deviation; TNM, tumor, node, metastasis system; DCIS, ductal carcinoma *in situ*; ER, estrogen receptor; PR, progesterone receptor; LVI, lymphovascular invasion; LA, luminal A; LB, luminal B; TNBC, triple-negative breast cancer.

^a^Kaplan–Meier univariate analysis of all factors.

^b^Adjusted by Cox proportional hazard regression model including all factors with the method of enter.

^c^Bold type indicates statistical significance.

^d^TNM stage is according to the 7th AJCC cancer staging system.

^e^Immunophenotype of invasive NPBC is according to the the immunohistochemical subtype of 2013 St. Gallen Consensus.

**Table 3:** Univariate and multivariate cox analysis of OS prognostic factors of screen-detected T1 invasive NPBC patients

Variables	Univariate^a^	Multivariate^b^	
	*P*^c^	HR (95% CI)	*P*^c^
**Age at diagnosis**	0.258	/	/
**Screening method**	0.526	/	/
**Accompanied with DCIS**	0.314	/	/
**Histological grade**	0.107	/	/
**pT**	0.144	/	/
**Lymph node status**	0.546	/	/
**pN**	**0.007**	/	/
**TNM stage^d^**	-	/	/
**Focality**	0.454	/	/
**LVI**	0.722	/	/
**ER status**	0.624	/	/
**PR status**	0.440	/	/
**Hormone receptor status**	0.670	/	/
**HER2 status**	0.620	/	/
**Ki-67 expression**	**0.001**	/	/
**p53**	0.488	/	/
**Immunophenotype**	0.383	/	/
**Luminal A**	0.147	/	/
**Luminal B**	0.143	/	/
**HER2**	0.139	/	/
**TNBC**	0.137	/	/
**Surgery**	0.389	/	/
**Chemotherapy**	0.791	/	/
**Radiotherapy**	**0.045**	**6.189 (1.064, 35.989)**	**0.042**
**Anti-Her2 targeted therapy**	0.778	/	/
**Endocrine therapy**	0.667	/	/

Abbreviations: NPBC, non-palpable breast cancer; US, ultrasound; MG, mammography; SD, standard deviation; TNM, tumor, node, metastasis system; DCIS, ductal carcinoma *in situ*; ER, estrogen receptor; PR, progesterone receptor; LVI, lymphovascular invasion; LA, luminal A; LB, luminal B; TNBC, triple-negative breast cancer.

^a^Kaplan–Meier univariate analysis of all factors.

^b^Adjusted by Cox proportional hazard regression model including all factors with the method of enter.

^c^Bold type indicates statistical significance.

^d^TNM stage is according to the 7th AJCC cancer staging system.

^e^Immunophenotype of invasive NPBC is according to the the immunohistochemical subtype of 2013 St. Gallen Consensus.

## DISCUSSION

Breast cancer incidence has considerably increased in the past three decades and is currently the most common cancer among Chinese women. The special concerns regarding breast cancer screening in China include the enormous population, the distinct epidemiology (majority of patients are pre-menopausal, early peak age around the 40s, small and dense breasts, etc.), and the rural-urban disparities in socioeconomic status and accessibility to medical resources [1, 2, 4, 10–14]. The currently mainstay screening modality in China is hospital-based opportunistic screening with ultrasound among self-referred asymptomatic women [3]. However, there was little information regarding the biological behavior and survival of these screen-detected NPBC in China.

Studies have shown that screen-detected breast cancer usually had low-risk tumor biology and favorable prognosis [6, 8, 15, 16]. Our study also showed the T1 NPBC had good 5-year short-term survival, and the OS of small breast cancer (T1a and T1b tumor) was 100%. Notably, 8.7% of T1a and 18.3% of T1b NPBC had positive lymph nodes, altogether 16.7% (35/210) overall incidence of lymph node (LN) metastasis all tumors < or =1 cm. This LN metastasis rate was higher than that of the study from Saiz et al., who reported that T1a breast cancer was unlikely to have demonstrable axillary lymph node metastases and lymph node dissections might be unnecessary. Yen TW et al. reported 80 out of 398 DCIS patients were found to have invasive cancer (mostly T1a disease) on final pathology, and 11 (11/80, 13.8%) patients who had positive sentinel lymph nodes (SLNs) manifested invasive cancer [17]. Francis AM et al. reported DCIS patients with occult invasion (mostly T1a cancer) and positive SLNs had the worse survival rate than pure DCIS regardless of positive SLNs [18]. There were 74 (71.8%, Supplementary Table 1) T1a patients had co-existing DCIS and micro-invasive, focal invasive and invasive disease in our study. Given the relatively high percentage of co-existing DCIS for screen-detected T1a NPBC, as well as the considerable incidence of LN metastasis in the T1a group, we suggest routine lymph node evaluation for these women.

In answer to the 3 questions in the Introduction, 1) LN metastasis potential significantly increased in parallel with the T1a, b, and c stage. Compared to T1a and b small breast cancer (≤ 1.0 cm), T1c tumor (1.1–2.0 cm) had less chance of being luminal A and more chance of TNBC. There was no significant difference in distribution of luminal B and Her2 subtype between T1 NPBC subgroups. Notably, the Ki-67 index also increased and the co-existing DCIS decreased significantly with T1 stage. This might suggest luminal A NPBC was more of a local disease, usually with co-existence of DCIS and lower proliferation rate at presentation, while triple-negative NPBC more of a systemic disease, scarcely accompanied with DCIS with higher proliferation rate.

2) For these small screen-detected invasive cancers, it was still the classical TNM stage and LN metastasis representing the early detection level that influences the survival as prognostic factors, rather than the subtype revealing the tumor biological nature that did. This might be due to the relatively short follow-up time and treatment choice. Although several large-scale studies had reported subtype as indicator for survival, this might be different for early and small screen-detected cancers [19, 20]. LN metastasis would be a key indicator of systemic potential for early detected disease such as NPBC. Concerning the relationship between LN metastasis and tumor size, Yu KD et al. presented a model to evaluate the metastatic potential (MP) by the difference of observed and expected number of involved LNs [21]. The expected number of involved LN was defined as tumor size/1.5, and the model was excellent for in T1–2 tumors with extensive LN involvement. According to this model, the metastatic potential of all T1a, b and a large portion of T1c NPBC could simply be reflected by the positive node number. This is coincided with our study results. Thus it might not be justified to over-treat a screen-detected T1c Her2 over-expressed or triple-negative LN negative invasive NPBC with, even in young Chinese patients.

3) Given the favorable prognosis, screen-detected T1 invasive NPBC could be considered as low risk cancer. LN metastasis, pN, TNM stage, LVI, PR status, Ki-67 index, chemotherapy and targeted therapy was identified as the DFS prognostic factors, whereas the pN, Ki-67 index and radiotherapy as the OS related predictors. These factors would help to differentiate the individualized risk of NPBC. Our study result was a little bit different from the study of Falck, A. K et al., who reported molecular subtype would help to define patients at low risk in screening [5]. This might be interpreted by the short follow-up time and the distinct epidemiology and screening background. Although the 5-year short-term predicted survival of the T1a, b NPBC was favorable, there was still 8.7% and 18.3% patients respectively with positive LN, and even 3.5% of T1b patients developed N3 disease. These patients should still be followed closely.

Our study had several limitations. Firstly, it was a retrospective single-center study with limited number of cases and relatively short follow-up time. There might be late recurrence after 5 years, especially for the luminal subtype, and the prognostic factors might be different. Secondly, majority of T1a NPBC was DCIS with micro-invasive and focal invasive cancer, and the biological behavior and prognosis might actually be representing the DCIS instead of the invasive cancer of much less tumor burden. Thirdly, the information of treatment choice was acquired from telephone call, computer prescription and the patients’ medical record. The patients’ compliance to treatment, especially to the endocrine therapy was not taken into account in this study. Fourthly, the DFS prognostic factors were identified by multi-variate Cox regression model including all factors with the method of enter, however, the OS related predictors with the method of forward stepwise due to very limited OS events.

In conclusion, the percentage of lymph node metastasis, pN, TNM stage, high Ki-67, receiving chemotherapy and targeted therapy increased in parallel with T1a, b, c unilateral invasive screen-detected NPBC. T1a disease had higher chance of being luminal A tumor and accompanied with DCIS whereas T1c tumor being triple-negative NPBC. The 5-year DFS and OS was favorable, and there was no significant difference in 5-year DFS or OS between the T1a, b, c subgroups or subtypes/immunophenotypes of T1 invasive NPBC. TNM stage and LN metastasis potential was identified as the DFS prognostic factors and radiotherapy as the OS predictor.

## PATIENTS AND METHODS

### Ethics statement

This study was approved by the Ethics Committee of the Peking Union Medical College Hospital, Chinese Academy of Medical Sciences.

### Patients and clinicopathological characteristics

From January 2001 to December 2014, 4,574 asymptomatic “screening positive” women (defined as BI-RADS 4 and 5 on imaging test) received biopsies in PUMC Hospital. These patients included self-referred women who came to PUMC Hospital for screening with positive findings, those transferred from other hospitals and healthcare institutions, and those from the community-based Two Cancer Screening Project in Beijing and in China [22–24] to PUMC Hospital as “screening positive” for biopsy or surgery (Figure 1). Approximately 1/3 of these patients were from Beijing, while the other 2/3 from other provinces in China.

729 NPBC were diagnosed out of the 4,574 biopsies, representing a total of 1.8–2.4 million asymptomatic women participated in the screening, estimated with the overall breast cancer incidence of 30–40/ten thousand in China [1]. All patients’ formalin-fixed paraffin-embedded (FFPE) pathological sections were reviewed to confirm the diagnosis. All NPBC patients were followed by telephone call and the out-patient clinics follow-up examinations records. 30 patients were excluded due to missing clinicopathological data, and 56 bilateral cancer patients were excluded due to possible bias on prognosis from contralateral cancer (Figure 1). The clinicopathological characteristics, treatment choice, 5-year DFS and OS were analyzed and compared among T1a, b, c and among subtypes/immunophenotypes of T1 unilateral invasive NPBC (Supplementary Table 1, Table 1, Figures 2, 3). The DFS and OS prognostic factors were identified by univariate and multivariate Cox analysis respectively (Tables 2, 3).

### Statistical analysis

The quantitative variables were compared with *t-test* and the categorical variables were compared with chi-square tests. Survival outcomes including 5-year predicted DFS and OS were analyzed and compared by the Kaplan–Meier curve method. Kaplan–Meier univariate analyses and Cox multivariate analyses were performed to identify the DFS and OS prognostic factors for all the T1 unilateral invasive NPBC. The significance threshold was set at *p* < 0.05. SPSS software, version 18.0 (SPSS, Inc. Chicago, IL, US) was used for all of the statistical analyses. The method of enter was used in multivariate Cox analysis for the identification of DFS factors while the method of forward stepwise was used for OS predictors due to the limited OS events.

## SUPPLEMENTARY MATERIALS FIGURES AND TABLES




