# Supplementary material for: Tumor biology, clinicopathological characteristics and prognosis of screen detected T1 invasive non-palpable breast cancer in asymptomatic Chinese women (2001–2014)
Source: Oncotarget. 2017 Feb 17;8(16):26221–30. doi: 10.18632/oncotarget.15431 (PMC5432251; doi:10.18632/oncotarget.15431)
Supplement: Supplementary file 1 [file oncotarget-08-26221-s001.pdf]

# Tumor biology, clinicopathological characteristics and prognosis of screen detected T1 invasive non-palpable breast cancer in asymptomatic Chinese women (2001–2014)

## Supplementary Materials

**Supplementary Table 1: Clinicopathological characteristics of screen-detected T1 invasive NPBC from asymptomatic Chinese women.** See Supplementary_Table_1
