# Supplementary material for: Tumor biology, clinicopathological characteristics and prognosis of screen detected T1 invasive non-palpable breast cancer in asymptomatic Chinese women (2001–2014)
Source: Oncotarget. 2017 Feb 17;8(16):26221–30. doi: 10.18632/oncotarget.15431 (PMC5432251; doi:10.18632/oncotarget.15431)
Supplement: Supplementary file 2 [file oncotarget-08-26221-s002.doc]

**Table 1. Clinicopathological characteristics of screen-detected T1 invasive NPBC from asymptomatic Chinese women**

| **Characteristics** | No. (%) of Patients (N=437) | | | P^a^ |
| --- | --- | --- | --- | --- |
|  | T1a NPBC  (N=103) | T1b NPBC  (N=142) | T1c NPBC  (N=192) |  |
| **Age (years)** |  |  |  | ­­­­ |
| **Mean±SD** | 49.8±11.155 | 52.88±13.466 | 50.22±11.918 | 0.079 |
| **Age group** |  |  |  | 0.159 |
| <40 | 13 (12.6) | 23 (16.1) | 32 (16.7) |  |
| 40~49 | 46 (44.7) | 41 (28.9) | 76 (39.6) |  |
| 50~59 | 23 (22.3) | 36 (25.4) | 46 (24.0) |  |
| ≥60 | 21 (20.4) | 42 (29.6) | 38 (19.7) |  |
| **Screening method** |  |  |  | **0.000** |
| US-detected | 77 (74.8) | 130 (91.5) | 178 (92.7) |  |
| MG-detected | 26 (25.2) | 12 (9.5) | 14 (7.3) |  |
| **Lymph node status** |  |  |  | **0.002** |
| Negative | 94 (91.3) | 116 (81.7) | 142 (74.0) |  |
| Positive | 9 (8.7) | 26 (18.3) | 50 (26.0) |  |
| **pN** |  |  |  | **0.028** |
| N0 | 94 (91.3) | 116 (81.7) | 142 (74.0) |  |
| N1 | 7 (6.8) | 17 (12.0) | 34 (17.7) |  |
| N2 | 2 (1.9) | 4 (2.8) | 7 (3.6) |  |
| N3 | 0 (0.0) | 5 (3.5) | 9 (4.7) |  |
| **TNM stage^b^** |  |  |  | **0.035** |
| Ia | 94 (91.3) | 116 (81.7) | 142 (74.0) |  |
| Ib | 0 (0.0) | 1 (0.7) | 0 (0.0) |  |
| IIa | 7 (6.8) | 16 (11.3) | 34 (17.7) |  |
| IIb | 0 (0.0) | 0 (0.0) | 0 (0.0) |  |
| IIIa | 2 (1.9) | 4 (2.8) | 7 (3.6) |  |
| IIIb | 0 (0.0) | 0 (0.0) | 0 (0.0) |  |
| IIIc | 0 (0.0) | 5 (3.5) | 9 (4.7) |  |
| **Accompanied with DCIS** |  |  |  | 0.000 |
| No | 29 (28.2) | 122 (85.9) | 175 (91.1) |  |
| Yes | 74 (71.8) | 20 (14.1) | 17 (8.9) |  |
| **Histological grade** |  |  |  | 0.056 |
| Low | 19 (18.4) | 37 (26.1) | 34 (17.7) |  |
| Medium | 48 (46.6) | 80 (56.3) | 110 (57.3) |  |
| High | 30 (29.2) | 19 (13.4) | 39 (20.3) |  |
| Unknown | 6 (5.8) | 6 (4.2) | 9 (4.7) |  |
| **Focality** |  |  |  | 0.514 |
| Monofocal | 81 (78.6) | 118 (83.1) | 161 (83.9) |  |
| Multifocal | 22 (21.4) | 24 (16.9) | 31 (16.1) |  |
| **LVI** |  |  |  | 0.730 |
| No | 99 (96.1) | 137 (96.5) | 182 (94.8) |  |
| Yes | 4 (3.9) | 5 (3.5) | 10 (5.2) |  |
| **ER** |  |  |  | 0.382 |
| Negative | 26 (25.2) | 24 (16.9) | 44 (22.9) |  |
| Positive | 77 (74.8) | 118 (83.1) | 147 (76.6) |  |
| Unknown | 0 (0.0) | 0 (0.0) | 1 (0.5) |  |
| **PR** |  |  |  | 0.115 |
| Negative | 37 (35.9) | 31 (21.8) | 58 (30.2) |  |
| Positive | 66 (64.1) | 111 (78.2) | 133 (69.3) |  |
| Unknown | 0 (0.0) | 0 (0.0) | 1 (0.5) |  |
| **Hormone receptor** |  |  |  | 0.326 |
| Negative | 20 (19.4) | 20 (14.1) | 42 (21.9) |  |
| Positive | 83 (80.6) | 122 (85.9) | 149 (77.6) |  |
| Unknown | 0 (0.0) | 0 (0.0) | 1 (0.5) |  |
| **Her2 status** |  |  |  | 0.061 |
| Negative | 69 (67.0) | 116 (81.7) | 151 (78.6) |  |
| Positive | 22 (21.4) | 20 (14.1) | 29 (15.2) |  |
| Unknown | 12 (11.6) | 6 (4.2) | 12 (6.2) |  |
| **Ki-67** |  |  |  | **0.017** |
| <14% | 59 (57.2) | 75 (52.8) | 78 (40.6) |  |
| ≥14% | 39 (37.9) | 65 (45.8) | 108 (56.2) |  |
| Unknown | 5 (4.9) | 2 (1.4) | 6 (3.2) |  |
| **p53** |  |  |  | 0.517 |
| Negative | 65 (63.1) | 104 (73.2) | 132 (68.8) |  |
| Positive | 36 (35.0) | 35 (24.5) | 55 (28.6) |  |
| Unknown | 2 (1.9) | 3 (2.1) | 5 (2.6) |  |
| **Immunophenotype ^c^** |  |  |  | 0.054 |
| Luminal A | 42 (40.8) | 62 (43.8) | 63 (32.8) |  |
| Luminal B | 31 (30.0) | 55 (38.7) | 79 (41.2) |  |
| Her2 | 10 (9.7) | 9 (6.3) | 15 (7.8) |  |
| TNBC | 8 (7.8) | 10 (7.0) | 25 (13.0) |  |
| Unknown | 12 (11.7) | 6 (4.2) | 10 (5.2) |  |
| **Luminal A** |  |  |  | **0.021** |
| Non-LA | 49 (47.6) | 74 (52.1) | 119 (62.0) |  |
| LA | 42 (40.7) | 62 (43.7) | 63 (32.8) |  |
| Unknown | 12 (11.7) | 6 (4.2) | 10 (5.2) |  |
| **Luminal B** |  |  |  | 0.077 |
| Non-LB | 60 (58.3) | 81 (57.0) | 103 (53.6) |  |
| LB | 31 (30.0) | 55 (38.8) | 79 (41.2) |  |
| Unknown | 12 (11.7) | 6 (4.2) | 10 (5.2) |  |
| **HER2** |  |  |  | 0.106 |
| Non-HER2 | 81 (78.6) | 127 (89.4) | 167 (87.0) |  |
| HER2 | 10 (9.7) | 9 (6.4) | 15 (7.8) |  |
| Unknown | 12 (11.7) | 6 (4.2) | 10 (5.2) |  |
| **TNBC** |  |  |  | **0.039** |
| Non-TNBC | 83 (80.5) | 126 (88.8) | 157 (81.8) |  |
| TNBC | 8 (7.8) | 10 (7.0) | 25 (13.0) |  |
| Unknown | 12 (11.7) | 6 (4.2) | 10 (5.2) |  |
| **Surgery** |  |  |  | **0.008** |
| Breast conserving surgery | 14 (13.6) | 42 (29.6) | 38 (19.8) |  |
| Mastectomy | 89 (86.4) | 100 (70.4) | 154 (80.2) |  |
| **Chemotherapy** |  |  |  | **0.000** |
| No | 82 (79.6) | 92 (64.8) | 82 (42.7) |  |
| Yes | 21 (20.4) | 50 (35.2) | 110 (57.3) |  |
| **Radiotherapy** |  |  |  | 0.207 |
| No | 89 (86.4) | 109 (76.8) | 150 (78.2) |  |
| Yes | 13 (12.6) | 33 (23.2) | 40 (20.8) |  |
| Unknown | 1 (1.0) | 0 (0.0) | 2 (1.0) |  |
| **Anti-Her2 targeted therapy** |  |  |  | **0.008** |
| No | 99 (96.1) | 122 (85.9) | 159 (82.8) |  |
| Yes | 3 (2.9) | 14 (9.9) | 29 (15.1) |  |
| Unknown | 1 (1.0) | 6 (4.2) | 4 (2.1) |  |
| **Endocrine therapy** |  |  |  | 0.402 |
| No | 20 (19.4) | 21 (14.8) | 42 (21.9) |  |
| Yes | 83 (80.6) | 121 (85.2) | 149 (77.6) |  |
| Unknown | 0 (0.0) | 0 (0.0) | 1 (0.5) |  |

Abbreviations: NPBC, non-palpable breast cancer; US, ultrasound; MG, mammography; SD, standard deviation; TNM, tumor, node, metastasis system; DCIS, ductal carcinoma in situ; ER, estrogen receptor; PR, progesterone receptor; LVI, lymphovascular invasion; LA, luminal A; LB, luminal B; TNBC, triple-negative breast cancer.

^a^ Bold type indicates statistical significance.

^b^ TNM stage is according to the 7^th^ AJCC cancer staging system.

^c^ Immunophenotype of invasive NPBC is according to the the immunohistochemical subtype of 2013 St. Gallen Consensus.
